# Electricity

**Published:** 1887-01

**Authors:** 


### ELECTRICITY.

### SINGULAR PHENOMENA OF THE NEW ZEALAND ERUPTIONS—A PUZZLING AFFAIR.

Among the many extraordinary natural phenomena attending the resent eruption of Mount Tarawera, one which appears to me not the least singular has been passed over in comparative silence and without exciting comment, so far as I am aware, among the scientific or unscientific public. During the last week those attending Mr. Burton's interesting lectures have heard there related one of the strange and, so far, inexplicable circumstances witnessed by Mr. McRea and others of that devoted little band to whom it must have seemed that hell itself had opened to destroy them. I allude to the fact of their being unable to make water boil on that terrible night, when earth itself appeared to be in a state of ebullition. I give here the narrative from Mr. McRea's own lips, and I feel confident that few who have read of the magnificent courage and presence of mind displayed by him among those fearsome surroundings, and none who have heard the plain, unvarnished tale modestly related by himself, will ascribe the circumstances as due to the working of an overheated and excited imagination.

Mr. McRea says: "I made George Baker, the cook, put some water on the fire to make cocoa for the women, who were cold and shivering, poor souls, though holding up grandly. About three-quarters of an hour afterward he met me in the passage and said to me: 'Come here, sir.' 'What is it?' said I. 'I can't get the water to boil,' he said. 'Tut,' said I, 'poke up the fire.' 'It's a good fire,' he replied, and so it was, a glowing one of blazing rata logs––a splendid fire. 'Put your hand in there and feel it,' said he, taking the lid off the boiler. I did so—very gingerly I can assure you, and found the water as cold as when we put it on. There were so many extraordinary things happening around me that this particular one did not excite my wonder very much. I thought it was owing to the electricity in the air. George Baker can vouch, as well as myself, for the fact of the water having been on the fire for full threequarters of an hour, and at the end of that time being as cold as when put on. We spoke of the circumstance to the others at the time as being curious, but soon had matters more serious to distract our attention."

Now, surely, here is a natural phenomenon worthy the investigation of all our scientific men, not only in New Zealand, but throughout the civilized world. We, of course, all know that the greater the atmospheric pressure the greater the number of units of heat required to make the water boil, but some other different cause must have been at work in this instance, as, after having been placed for three-quarters of an hour on a good fire, the water remained absolutely cold. What other cause was there? is the problem I suggest to our scientific men as one well worthy of their research.—*Cor. New Zealand Herald.*

---

It is presumed that the overcharge of electricity which pervaded the atmosphere for the time being, used up the caloric generated by the fire as rapidly as it was produced, leaving none for household purposes. In a word, electricity played the part of the capitalist, and while contributing liberally to the fire, made way with the profits as fast as accumulated, with no object of establishing anything like an equilibrium.—Ed.

